# Cerebral arterial air embolism after computed tomography-guided hook-wire localization of a pulmonary nodule: A case report

**DOI:** 10.1097/MD.0000000000015437

**Published:** 2019-05-03

**Authors:** Ming-Yi Wang, Yong-Sheng Liu, Xiang-Bo An, Ke Li, Yong-Jian Liu, Feng Wang

**Affiliations:** Department of Intervention Therapy, First Affiliated Hospital of Dalian Medical University, Dalian, China.

**Keywords:** air embolism, complication, computed tomography-guided intervention, hook-wire localization

## Abstract

**Rationale:**

Cranial arterial air embolism is a rare but potentially fatal complication after computed tomography (CT)-guided pulmonary interventions.

**Patient concerns:**

A 64-year-old man was diagnosed with a pulmonary nodule (diameter: approximately 1 cm) in the right lower lobe. The patient developed convulsions after CT-guided hook-wire localization.

**Diagnosis:**

Cranial CT revealed arborizing/linearly distributed gas in the territory of the right middle cerebral artery.

**Interventions:**

The patient was administered hyperbaric oxygen, antiplatelet aggregation therapy, and dehydration treatment.

**Outcomes:**

Clinical death occurred 55 hours after air embolism.

**Lessons:**

Systemic air embolism is a serious complication of lung puncture. Clinicians should improve their understanding of this complication and remain vigilant against air embolism.

**KEY POINTS**

- Air embolism is a rare but serious complication of hook-wire localization. We present a rare case of cerebral arterial air embolism after computed tomography (CT)-guided lung puncture and hook-wire localization. The clinical features and treatment experiences of the patient are described.
- This study analyzes in detail the possible mechanisms by which gas enters the pulmonary veins.

## 1 Introduction

The diagnosis of nodular pulmonary lesions (NPLs) of unknown origin is sometimes challenging, and a subset of NPL is not suitable for transthoracic needle biopsy. Video-assisted thoracoscopic surgery (VATS) can be used for simultaneous diagnosis and resection of NPL. It is an effective method for diagnosis and treatment of NPL owing to its minimally invasive nature. However, most NPLs are small and located deep in the intrapulmonary tissue, and difficult to localize accurately during VATS thereby reducing its success. The preoperative computed tomography (CT)-guided hook-wire localization technique was 1st proposed by Mack et al.^[1]^ However, hook-wire localization is an invasive procedure associated with a high risk of complications. Air embolism is a rare but serious complication of hook-wire localization. We present a rare case of cerebral arterial air embolism after CT-guided lung puncture and hook-wire localization. The clinical features and treatment experiences of the patient are described.

## 2 Case report

**Figure 1:**
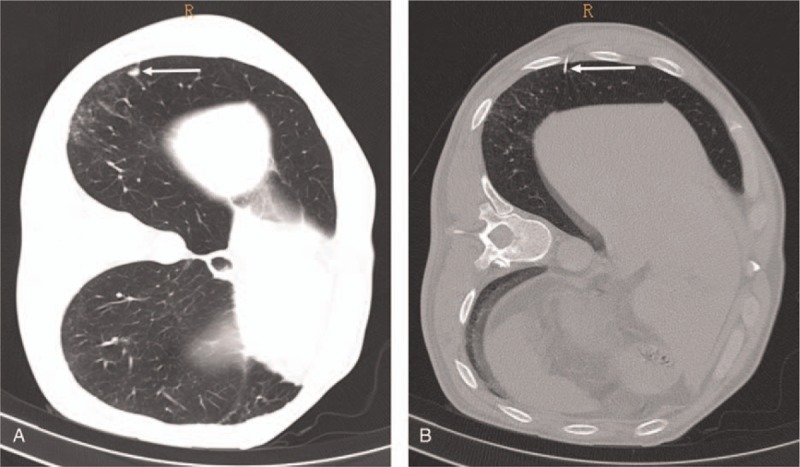
Preoperative computed tomography showing a right pulmonary nodule (A) and intraoperative puncture location (B).

A 64-year-old man with no significant past medical history was diagnosed with a pulmonary nodule (diameter: approximately 0.7 cm) in the right lower lobe 2 years ago, the patient did not follow the doctor's advice to perform chest CT scan after 6 to 8 months. In a recent health check-up, chest CT showed increase in size of the nodule to 1 cm (Fig. 1A). VATS wedge resection of right lower lobe was planned. CT-guided hook-wire localization of the lung lesion under local anesthesia was planned before surgery using a breast localization needle (21 gauge; AccuraBLN2110, Angiotech Biomaterials, Palo Alto, CA) owing to the small size of the nodule.

**Figure 2:**
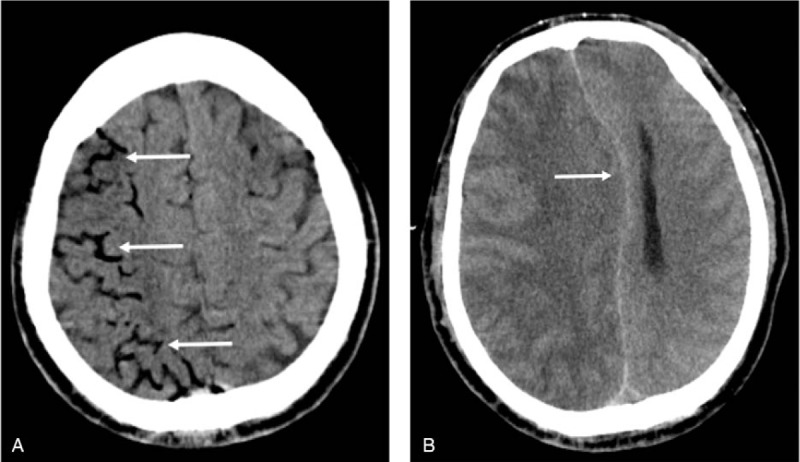
(A) Right middle cerebral artery branches with massive gas. (B) Cranial computed tomography showing large cerebral infarction in the right hemisphere and subfalcine herniation 48 hours after air embolism.

The patient was placed in the left lateral decubitus position. After a positioning scale was attached to the chest wall surface, thin-section CT scan was performed to determine the puncture point and direction. The skin around the puncture point was disinfected and draped followed by injection of 2% lidocaine (5 mL) for local infiltration anesthesia. Percutaneous lung puncture was performed according to the predetermined puncture point and direction, and a 10-cm needle was quickly inserted into the lung tissue while the patient held his breath at the end of inspiration. The proper location of the needle was confirmed by a thin-section CT scan. The positioning guide wire was then deployed, the puncture needle was removed, and the guide wire was gently pulled outwards to fix the barb at its front end to the lung tissue (Fig. 1B). Unexpectedly, the patient suddenly lost consciousness and suffered convulsions during the pulling process. Physical examination showed: light coma, GCS score of 13 points; the pupils were symmetrical (diameter 3 mm) and reactive to light; there was no neck rigidity; limb twitching was present; and positive Babinski sign was observed on both sides. Emergency cranial CT revealed arborizing/linearly distributed gas in the territory of the right middle cerebral artery (MCA), which was suggestive of air within the cortical branches of the right MCA (Fig. 2A). Chest CT showed no signs of pneumothorax or any obvious abnormality of large cardiac vessels. Therefore, the patient was diagnosed with cerebral arterial air embolism. Medical treatment was administered, including hyperbaric oxygen and antiplatelet aggregation therapy. However, there was no improvement in the patient's condition. Brain CT reexamination performed 24 hours after air embolism showed disappearance of gas densities and a large infarct in the territory of right MCA with diffuse brain edema in the right hemisphere. Dehydration treatment was administered to reduce intracranial pressure; however, no improvement in the patient's condition was observed. Forty-eight hours after air embolism, cranial CT revealed aggravated cerebral edema, a marked shift of the midline structures, and subfalcine herniation (Fig. 2B). The patient's consciousness disturbance aggravated and the patient slipped into deep coma. The pupils were bilaterally asymmetrical and did not react to light. The patient exhibited increased difficulty in breathing. His legal guardian refused decompressive craniectomy or ventilatory support. Clinical death occurred 55 hours after air embolism.

## 3 Discussion

In this report, we present a case of cerebral artery air embolism after CT-guided hook-wire localization. Systemic air embolism is a rare complication of lung biopsy/intervention, with a reported prevalence of 0.02% to 0.07%.^[2,3]^ The incidence rate of this complication after CT-guided hook-wire localization is lower than that after puncture biopsy due to the larger bore of the biopsy needle and different operative methods for these 2 techniques. Only a few cases of air embolism occurring after CT-guided hook-wire localization have been reported.^[4–6]^ Important complications of this procedure include pneumothorax, pulmonary hemorrhage, and air embolism. Air embolism is the most serious complication because of its quick onset, severe symptoms, and high mortality.

Systemic air embolism occurs due to entry of gas in the systemic circulation from the pulmonary vein through the left atrium and left ventricle. There are 3 possible mechanisms for the entry of gas into the pulmonary veins. Firstly, extrapulmonary air may enter the pulmonary vein directly via the puncture needle. After the needle pierces the pulmonary vein, the needle core is pulled out and the needle hub is directly exposed to the air. This forms a passage between the outside air and pulmonary vein. On deep inhalation by the patient, air is liable to enter the pulmonary vein through the puncture needle because pulmonary venous pressure is lower than the atmospheric pressure at this time. In the present case, the possibility of direct communication between the outside air and the pulmonary vein during the procedure of CT-guided hook-wire localization is extremely low due to the special structure of the hook-wire puncture system and the operation methods. Secondly, gas may enter the pulmonary vein from the pulmonary artery through pulmonary microcirculation or pulmonary arteriovenous fistulas. However, lung puncture rarely causes the entry of gas into the pulmonary artery.^[7]^ Thirdly, intrapulmonary air may enter the pulmonary vein through the puncture channel. Passage of the puncture needle through the lung cavity, bronchus, or the normal alveolar tissue may injure the adjacent pulmonary veins, which forms a communication between the airway and the pulmonary vein. Valsalva movements or coughing may render the intrapulmonary pressure higher than the pulmonary venous pressure, which facilitates the entry of air into the pulmonary veins through the puncture channel. Under normal circumstances, pulmonary vein injury induces vasoconstriction and initiates intravascular and external coagulation, which eventually leads to occlusion of the damaged vessels. However, in patients with infection at the puncture site, vasculitis, pulmonary fibrosis, or coagulation dysfunction, the normal coagulation mechanism is impaired, which delays the vessel wound closure.^[7]^ After the lung tissue is damaged by puncture needle, the wound tends to close due to elastic retraction. Pleural adhesions, lung tissue consolidation, or fibrosis at the puncture site reduces lung compliance, impairs elastic retraction, and delays the closure of the puncture channel. These mechanisms provide pathologic anatomical basis for the occurrence of air embolism.^[8]^

In the present case, the most likely mechanism was the entry of intrapulmonary air into the pulmonary vein through the puncture channel. Although preoperative chest CT did not reveal any lung cavity, infection, fibrosis, or pleural adhesions, severe air embolism still occurred. A probable explanation is that the puncture needle pierced the adjacent pulmonary vein and bronchus at the same time; when the guide wire was released and the needle pulled outwards, the front barb of the guide wire hooked on to the bronchus and the pulmonary vein to cause an abnormal anastomosis, equivalent to that observed in a continuous bronchopulmonary vein fistula.

In the meantime, the patient's deep inspiration may have led to a decrease in the pulmonary vein pressure. Consequently, a large volume of air in the lung tissues passed through the fistula and entered the pulmonary veins, and then entered the systemic circulation through the left heart system. The patient was in the left lateral decubitus position. Therefore, the gas was more likely to have entered the brachiocephalic artery and the right internal carotid artery.

The pathophysiologic changes caused by air embolism mainly include ischemia (caused by direct gas embolization), intense vasospasm (due to stimulation by gas embolus), and platelet-activated microthrombi (caused by vascular endothelial injury).^[9]^ Clinical manifestations depend on the embolized blood vessels.^[7]^ Small air embolus has little effect on skeletal muscles and abdominal organs, and generally does not lead to obvious clinical symptoms related to these organs.^[9]^ However, a small amount of gas embolus in the cerebrovascular or coronary artery can cause serious symptoms and even death.^[7]^

Air embolism is easily missed in the absence of any overt symptoms. Brain and chest CT can be used to confirm the presence of gas in the cerebral artery, pulmonary vein, left atrium, left ventricle, aorta, or coronary artery. Kuo et al^[10]^ proposed that the scope of the CT scan after lung puncture should include the heart and aortic arch to detect asymptomatic air embolism and to facilitate prompt treatment.

Hyperbaric oxygen therapy decreases the volume of air bubbles, increases the oxygen solubility in the blood, promotes the release of nitrogen in the air bubble into the blood, attenuates vascular permeability, and reduces vascular endothelial damage. It is currently recognized as the 1st-line therapy for the treatment of systemic air embolism.^[11]^ Early hyperbaric oxygen therapy improves prognosis of patients with cerebrovascular air embolism and reduces their mortality rate to 7%.^[12]^ The present patient was promptly administered hyperbaric oxygen therapy. However, a large cerebral infarct still occurred. This is likely attributable to massive air embolization of the trunk and branches of intracranial arteries and poor compensatory collateral circulation which led to brain cell necrosis and edema in a short time.

Systemic air embolism is a rare and serious complication of lung puncture. It is characterized by quick onset, severe symptoms, and high mortality. Clinicians should improve their understanding of this complication and remain vigilant against air embolism, and discover and treat this complication early to reduce its sequelae and mortality.

## Author contributions

MYW and FW wrote the manuscript; YSL contributed the central idea; XBA and KL contributed to the interpretation of results; YJL revised the manuscript.

**Conceptualization:** Ming-Yi Wang, Yong-Sheng Liu, Xiang-Bo An.

**Data curation:** Ming-Yi Wang.

**Formal analysis:** Ming-Yi Wang, Ke Li.

**Funding acquisition:** Xiang-Bo An, Yong-Jian Liu.

**Methodology:** Yong-Sheng Liu, Xiang-Bo An.

**Project administration:** Yong-Sheng Liu, Xiang-Bo An.

**Resources:** Yong-Sheng Liu, Ke Li, Yong-Jian Liu, Feng Wang.

**Software:** Ke Li, Yong-Jian Liu, Feng Wang.

**Supervision:** Ke Li, Feng Wang.

**Validation:** Ke Li, Feng Wang.

**Visualization:** Feng Wang.
